# Blind Interference Suppression with Uncalibrated Phased-Array Processing †

**DOI:** 10.3390/s25072125

**Published:** 2025-03-27

**Authors:** Lauren O. Lusk, Joseph D. Gaeddert

**Affiliations:** Virginia Tech National Security Institute, Blacksburg, VA 24061, USA; jgaedder@vt.edu

**Keywords:** beamforming, digital phased arrays, interference suppression, singular value decomposition

## Abstract

As the number of devices using wireless communications increases, the amount of usable radio frequency spectrum becomes increasingly congested. As a result, the need for robust, adaptive communications to improve spectral efficiency and ensure reliable communication in the presence of interference is apparent. One solution is using beamforming techniques on digital phased-array receivers to maximize the energy in a desired direction and steer nulls to remove interference; however, traditional phased-array beamforming techniques used for interference removal rely on perfect calibration between antenna elements and precise knowledge of the array configuration. Consequently, if the exact array configuration is not known (unknown or imperfect assumption of element locations, unknown mutual coupling between elements, etc.), these traditional beamforming techniques are not viable, so a beamforming approach with relaxed requirements (blind beamforming) is required. This paper proposes a novel blind beamforming approach to address complex narrowband interference in spectrally congested environments where the precise array configuration is unknown. The resulting process is shown to suppress numerous interference sources, all without any knowledge of the primary signal of interest. The results are validated through wireless laboratory experimentation conducted with a two-element array, verifying that the proposed beamforming approach achieves a similar performance to the theoretical performance bound of receiving packets in additive white Gaussian noise (AWGN) with no interference present.

## 1. Introduction

### 1.1. Problem Overview

With the increased demand for robust and reliable wireless communications links, open spectrum bands are becoming increasingly scarce, particularly as spectrum sharing solutions become more prevalent. Spectrum sharing solutions are especially of interest in the industrial, scientific, and medical (ISM) bands. Historically, these bands are reserved for devices such as microwave ovens and shortwave medical heating devices. However, since the Federal Communications Commission (FCC) opened the bands to unlicensed users in 1989 [1], a growing number of both licensed and unlicensed users have attempted to share the available spectrum. Many of the unlicensed users are short-ranged, low-power wireless communication systems that take advantage of the unlicensed spectrum to cut costs. These unlicensed devices include Bluetooth, contactless smart cards, wireless microphones, garage door openers, radio frequency identification (RFID) tags, Wi-Fi, and other devices associated with the Internet of Things (IoT). Although they are permitted to transmit in the bands, unlicensed devices are not allowed to interfere with the transmission of licensed devices and must accept a certain amount of interference. As the number of devices using the ISM bands increases, so must the amount of interference a device must contend with.

Furthermore, as shared spectrum bands incorporate more and more disparate signal types and waveforms, new solutions need to account not only for the interference they receive but also for the interference they impart on existing users. For the general problem of robust wireless communications in congested spectrum environments, reliability can be achieved in several different ways:

- Spread-spectrum communications which generally distribute the information rate disproportionately across the bandwidth so as to minimize the statistical impact of interference;
- Successive interference cancellation which seeks to iteratively remove interference through estimation and subtraction;
- Spectral white-space estimation methods which attempt to locate (and possibly predict) which frequency bands are available, often using non-contiguous spectrum waveforms;
- Multi-antenna methods which include an entire array of techniques to provide spatial diversity.

Combinations of the above can also be used to improve reliability and mitigate interference; however, the combined techniques are often still restricted by the limitations of the underlying techniques. Spread-spectrum techniques accept a certain amount of performance degradation on both the legacy and secondary users while successive interference cancellation methods are computationally intense and rely heavily on the correct estimation of parameters to operate. Likewise, white-space estimation requires waveform re-definition, and multi-antenna methods traditionally require precise calibration to produce accurate beams (e.g., nulls in the direction of interference sources).

The potential benefit in spatial diversity afforded by multi-antenna systems is substantial in its ability to simultaneously null interference while providing gain in the direction of a desired signal. In particular, digital phased-array systems provide substantial benefits over their analog counterparts, including the ability to process simultaneously occurring distinct beams and apply any number of signal processing techniques for multi-spatial signal separation. Traditional digital phased-array processing, however, is expensive and requires precise calibration of antenna spacing to properly steer beams. The calibration issue becomes increasingly complex when modern platforms are taken into account. Many modern platforms are often already overloaded with existing diverse and possibly heterogeneous antennas and have little to no space to incorporate a large array. As such, an optimal solution would be to treat the existing antennas as a digital array; however, the calibration between the existing antennas might not be precise enough to utilize traditional beamforming algorithms. Additionally, narrowband interfering signals may be too numerous for the degrees of freedom afforded by the array to null; this is particularly true for recovering a wideband carrier in congested frequency bands.

### 1.2. Traditional Beamforming Methods

Traditional phased-array processing treats the combination of signal of interest (SOI) and interfering signals as a linear combination of vectors of length *N* (which corresponds to the number of sensor elements in the array) over time and attempts to maximize the output signal-to-interference-plus-noise ratio (SINR) by steering a beam in the direction of SOI as it points nulls in the direction of interferers. An array with *N* elements has N−1 degrees of freedom, so it is only able to guarantee controlling at most N−1 nulls, which means the array can only guarantee the removal of at most N−1 interfering signals.

Assuming the precise array configuration and the direction of arrival (DOA) of SOI are known a priori, the minimum variance distortionless response (MVDR) beamformer is considered an optimal solution [2]. This type of beamforming creates weights that minimize the amount of interference and noise while maintaining a distortionless response in the DOA of SOI, thus maximizing the SINR of the output. However, if the knowledge of SOI’s DOA or the array configuration is incomplete or imprecise, the performance will degrade as SOI is treated as interference instead of as the desired signal. Similarly, if either the DOA of SOI or the array configuration is unknown, MVDR cannot be used. While MVDR’s limitations are historically well documented and addressed in the literature [3,4], it is not always feasible to know SOI’s DOA a priori, so DOA estimation techniques such as multiple signal classification (MUSIC) are employed. However, MUSIC has its own set of requirements as a subspace-based algorithm (e.g., accurate covariance matrix estimation, known number of signal sources (both interference and SOI), all received signals are uncorrelated), which might preclude it from being a viable option [5,6,7]. As such, blind beamforming methods are of primary interest.

Blind beamforming methods assume no knowledge of the DOA of any signal (including SOI as well as the interfering signals). They seek to adapt the beam weights to isolate SOI, whose characteristics may still be unknown; however, like with traditional approaches, the array configuration and phase alignment of the elements are still known a priori. Generally, blind approaches can be assigned to one of three categories: statistics-based, eigen-based, and deep learning approaches. Statistics-based approaches require some statistical characteristic of SOI to be known a priori, such as a constant modulus for constant modulus algorithm (CMA) [8,9,10,11], cyclosationary properties for spectral self-coherent restoral (SCORE) [12,13,14], and a non-Gaussian distribution [15]. Though effective, these approaches will not work if SOI does not possess the specific statistical characteristic required for the algorithms, and the algorithms also still rely on perfect knowledge of the array configuration to properly beamform.

Eigen-based beamformers are naturally more robust against an imperfectly assumed array configuration. This group of beamformers typically decomposes a covariance matrix (consisting of some combination of interference, signal, and noise) into its eigenvalues and eigenvectors to identify a composite steering vector (CSV), with which to steer the beamformer [16,17,18]. Thus, while eigen beamformers do not rely upon knowledge of the DOA, precise knowledge or accurate estimation of the covariance matrix is essential. It is also important to note that if any coherent signals do exist, the rank of the source covariance matrix is reduced, which in turn renders the majority of the eigen-based beamformers ineffective. In such a case, which can be typical of multipath environments, additional processing must be used such as spatial smoothing [19]. Finally, like the previously mentioned beamforming algorithms, eigen-based beamformers are still limited to being able to only null as many interfering signals as there are degrees of freedom.

Finally, with gaining popularity, deep learning methods are used to determine the optimal beamforming weight vector. A few reasons to account for their growing popularity include the ability to run parallel processing to suppress wideband interference and remove the reliance on DOA [20,21,22]. Although these approaches offer solutions for many issues associated with traditional and blind beamforming (precise knowledge of DOA, knowledge of the interference covariance matrix, slow convergence, etc.), deep learning introduces new concerns such as the development of training data. As such, deep learning approaches are not considered. Instead, this paper proposes a non-deep learning, subspace-based approach that is not reliant upon precise DOA or array configuration knowledge, an accurate covariance matrix, or the statistical properties of SOI while also introducing more degrees of freedom.

### 1.3. Non-Beamforming Methods

One non-beamforming method for interference suppression is successive interference cancellation (SIC) [23]. Generally speaking, if two or more signals are received concurrently, the strongest (highest SINR) is demodulated and decoded then subtracted from the aggregate received signal. The resulting residual only contains the weaker signal or signals. If there were more than two signals in the aggregate received signal, then the process is repeated with the residual until only the weakest signal is left. Since this process is successive, it has the potential to perfectly remove interfering signals; however, if one of the signals is estimated incorrectly, all of the subsequently estimated signals will also be incorrect and the error will grow with each iteration. This means that if the interference cannot be estimated (as is the case with uncorrelated noise-like interference sources), SIC methods are not effective and can actually degrade performance. As such, these techniques are not considered in this paper.

Another non-beamforming method used for interference suppression is space–time adaptive processing (STAP). This type of processing is particularly useful for frequency-selective channels, can combat multipath fading, and suppresses both intersymbol interference (ISI) and co-channel interference (CCI) [24]. As defined by [25], the primary goal of STAP is to increase the dimensions of the covariance matrix while reducing the spatial correlation between the steering vectors of the received signals. In their paper, the authors propose an STAP approach (based on MVDR) to estimate the DOA of a particular signal by introducing time delay taps at each sensor and thus expanding the dimensions of the received data matrix. This method is proven in simulation to have higher spatial resolution than MVDR for waveforms with a low signal-to-noise ratio (SNR) (−5 dB) and an array with few degrees of freedom (eight elements). However, since STAP is a type of adaptive processing, the time it takes to converge can be a concern. Additionally, as the data rate, delay profile, or number of sensors increases, the complexity of STAP increases sharply. One method to combat the increasing complexity is to implement STAP on sub-bands. By introducing sub-bands, STAP can be implemented in parallel, converge rapidly, and have a reduced processing complexity [26]. While the issue of increased complexity and slow convergence can be mitigated by using sub-bands, the method provided by [26] is only proven under the assumption that all of the received signals (including both SOI and interferers) are wide-sense stationary and that the number of signals present is less than the number of sensors in the array.

Furthermore, while the design of new waveforms can partially address this calibration problem, many legacy systems are inflexible to changes to the physical layer outside the radio frequency front end (RFFE). As such, this paper proposes singular value interference removal algorithm (SVIRA), a method capable of supporting legacy systems operating in new environments, rather than designing a new waveform to accommodate the specific demands of the user. The remainder of this paper is organized as follows: Section 2 provides an overview of the proposed algorithm, Section 3 describes the laboratory setup and experimentation, Section 4 gives an overview of the results and findings, and Section 5 provides some concluding remarks.

## 2. Materials and Methods

### 2.1. Overview

The general problem of multi-antenna interference suppression involves detecting the presence of one or more nuisance signals and suppressing their energy while amplifying an intended SOI using the spatial separation of sensor elements. Let us first consider a wideband SOI, represented as a discrete set of *K* band-limited linear modulated symbols at a baud rate of 1/T, viz.

(1) $$s\left(t\right)=\sum_{k=0}^{K-1}a_{k}g\left(t-kT\right)$$

where $a_{k}\in A$ denotes a set of uncorrelated and unknown data symbols and g(t) is a band-limited square-root Nyquist pulse-shaping filter. For this problem, it is assumed that all values of *K*, $a_{k}$, A, g(t), and *T* are unknown; however, the power spectral density (PSD) of s(t) can be assumed to be reasonably flat and occupying a considerable portion of the sampled bandwidth.

The receiver is a collection of *N* sensing antennas with an unknown spatial configuration. The received signal is a delayed version of s(t) with a carrier frequency, phase offset, and channel gain (due to path loss) and is distorted by a set of interfering signals. The continuous-time representation of the received complex baseband signal on sensor *i* can be represented as

(2) $$r_{i}\left(t\right)=\gamma s\left(t-\tau \right)e^{j\alpha_{i}}+\sum_{p}\zeta_{p}\left(t\right)e^{j\beta_{i,p}}+w_{i}\left(t\right),$$

where $\zeta_{p}\left(t\right)$ is one of *P* unknown interfering signals, γ is an unknown but fixed channel gain, and $w_{i}\left(t\right)$ is a wide-sense stationary random Gaussian process with a PSD of $N_{0}/2$. It should be noted that all of the interfering signals are assumed to be narrowband and may be spatially separated. Also, SOI is assumed to be uncorrelated with both the interfering signals and noise. Furthermore, while the exact noise PSD is not known, it can be estimated. Finally, the received signal from each sensor then passes through a discrete-time sampler at a rate of $F_{s}=1/T_{s}$. The final discrete-sampled time series on sensor *i* at time index *n* is thus

(3) $$r_{i}\left(n\right)=r_{i}\left(nT_{s}\right)$$

An array with *N* sensors has N−1 degrees of freedom for suppressing interference signals in the band. For environments where the number of nuisance signals exceeds *N*, the array cannot effectively suppress this interference and will perform poorly; however, assuming nuisance signals will most likely not overlap in frequency, the received signals may be partitioned into orthogonal spectral sub-bands for further analysis. This reduces the number of nuisance signals seen by any one sub-band, allowing for better spectral discrimination. Once the interference removal has been completed, the wideband signal must be reconstructed from its interference-mitigated constituents. Perfect reconstruction for a function F and its inverse $F^{-1}$ are for the output to match its input, outside of a scale and time shift, viz.

(4) $$\begin{array}{ccc} \hat{x}\left(n\right) & = & F^{-1}\left(F\left(x(n)\right)\right) \end{array}$$

(5) $$\begin{array}{ccc} & = & cx(n-k) \end{array}$$

for a scaling constant *c* and fixed delay *k* ([27], p. 132). There are several possibilities for this processing. Perhaps the first that comes to mind is the fast Fourier transform (FFT) and its inverse which easily satisfies (5); however, the spectral performance of an FFT is very poor, exhibiting a 1/f rolloff and only a 13 dB side-lobe suppression with neighboring channels.

We propose the use of a polyphase filterbank (PFB) channelizer as a method to decompose the spectrum into narrowband orthogonal components. PFB channelizers can perform this computation efficiently when certain constraints are met (e.g., even spacing between the channels and identical resampling rate for each of the channels) through the use of a single prototype filter and FFT. By allowing the output rate to exceed the maximally decimated rate, and with care taken in designing the prototype filter, the output of the analysis channelizer can be fed into its complementary synthesis channelizer to provide an almost-perfect reconstruction of the input. In general, a true-perfect reconstruction is not possible with finite impulse response channelizers using a prototype filter; however, a near-perfect reconstruction with a slight delay and amplitude variation can be achieved by oversampling.

Oversampling by a factor of two allows for a simple architecture for processing signals. To make the channelizer oversample by a factor of 2, the analysis filterbank takes in M/2 samples instead of the usual *M* [28]. With sufficient care for the selection of channelizer parameters, both the number of nuisance signals observed within any one sub-band is within the degrees of freedom of the sensor array to suppress, and the number of samples on the output in each sub-band provides sufficient information for SVD factorization.

### 2.2. Analysis Polyphase Filterbank Channelizers

The analysis channelizer takes the received signal from the *i*-th antenna element $r_{i}\left(n\right)$ and outputs *M* signals $R_{i}\left(n,m\right)$, where *m* is the channel band number (m=0,1,…,M−1), using an analysis polyphase filterbank. It is so named because it “analyzes” the spectral components of $r_{i}\left(n\right)$. If we say a polyphase filter’s impulse response is as defined in [29] (11.10.10), then

(6) $$h_{i}\left(n,m\right)=h_{0}\left(nD-m,m\right),\;m=0,1,\ldots ,M-1$$

where *D* is the decimation factor and $h_{0}$ is the impulse response of the prototype filter. In this paper, the prototype filter is a sinc function with a Kaiser–Bessel window with a semi-length of 12 and a side-lobe suppression of 60 dB. Based on the polyphase filter impulse response, the decimated input sequence can be expressed as

(7) $$r_{i}\left(n,m\right)=r_{i}\left(nD+m,m\right)\;m=0,1,\ldots ,M-1$$

It should be recalled that the output of a general uniform discrete Fourier transform (DFT) filterbank is

(8) $$\begin{array}{c} R_{i}\left(k,m\right)=\left[\sum_{n}r_{i}\left(n,m\right)h_{0}\left(kD-n,m\right)e^{j2\pi m(kD-n)/M}\right]e^{-j2\pi kmD/M} \end{array}$$

where *k* is the index of the decimated samples and $R_{i}\left(\cdot \right)$ denotes the frequency domain. It is noticed that the summation inside the bracket computes the DFT of the filtered $r_{i}\left(n,m\right)$, and the exponential shifts the resulting DFT down to a complex baseband. Using (8), the output of the polyphase filter is

(9) $$R_{i}\left(k,m\right)=\sum_{m=0}^{M-1}\left[\sum_{l}h_{i}\left(l,m\right)r_{i}\left(k-l,m\right)\right]e^{-j2\pi nmD/M}$$

for m=0,1,…,M−1. It is observed that the summation inside the brackets represents the convolution of $r_{i}\left(l,m\right)$ with the impulse response $h_{i}\left(l,m\right)$, and the summation outside of the brackets computes the DFT of the filtered $r_{i}\left(l,m\right)$. As seen by the bounds of the outer summation, this does indeed result in the desired *M* samples. It is important to note that for the SVIRA proposed in this paper, the analysis filterbank outputs at 2/M the input rate or twice that of a maximally decimated channelizer (D=M/2).

### 2.3. Synthesis Polyphase Filterbank Channelizers

Designing a synthesis polyphase filter is a similar process to designing an analysis polyphase filter but effectively with all of the processing carried out in reverse. The synthesis filter is so named because it combines the individual components and “synthesizes” a wideband signal. First, the inverse DFT must be taken. Then, the signals are up-sampled through an interpolation filter. Finally, all of the signals are combined back into one. This process can be expressed mathematically in a uniform DFT case as given by [29] (11.10.9)

(10) $$v_{i}\left(n\right)=\frac{1}{M}\sum_{k=0}^{M-1}\left\{\sum_{m}\left[Y_{i}\left(k,m\right)e^{j2\pi kmI/M}\right]g\left(n-mI,m\right)\right\}$$

where *I* is the interpolation factor, which is equal to *D*, and g(n,m) is the impulse response of the interpolation filter

(11) $$g\left(n,m\right)=g_{0}\left(n\right)e^{j2\pi nm/M}$$

where $g_{0}\left(n\right)$ is the impulse response of the prototype filter. For the polyphase case, a similar impulse response for the interpolation filter is determined

(12) $$q_{i}\left(n,m\right)=g_{0}\left(nI+m\right)\;m=0,1,\ldots ,M-1$$

noting that the impulse response of the synthesis prototype filter $g_{0}\left(n\right)$ does not necessarily relate to that of the analysis prototype filter $h_{0}\left(n\right)$. Since D=I=M/2 in this paper, (10) can be rewritten as

(13) $$\begin{array}{cc} v_{i}\left(n,l\right) & =\sum_{m}q_{i}\left(n-k,l\right)\left[\frac{1}{M}\sum_{k=0}^{M-1}Y_{i}\left(k,m\right)e^{j2\pi ml/M}\right] \end{array}$$

(14) $$\begin{array}{cc} \; & =\sum_{k}q_{i}\left(n-k,l\right)y_{i}\left(k,l\right) \end{array}$$

for l=0,1,…,M−1. The simplification between (13) and (14) is possible by recognizing that the portion within the brackets in (13) is the inverse DFT of $Y_{i}\left(k,m\right)$, and thus, everything in the brackets can be replaced by $y_{i}\left(k,l\right)$, which is the inverse DFT of $Y_{i}\left(k,m\right)$. For more information on polyphase filters, see [29].

### 2.4. Almost-Perfect Reconstruction

Satisfying the requirement in (5) is possible with PFB channelizers with M>2, but only when individual band-pass filters are used for each channel [27]. Perfect reconstruction is not possible with DFT-based PFB channelizers with M>2 and can be impractical for most applications; however, almost-perfect reconstruction is vastly easier to implement despite adding negligible distortion. Almost-perfect reconstruction is possible by eliminating aliasing between sub-channels by

1. suppressing non-adjacent channels;
2. canceling aliasing between the overlapping pass-bands of adjacent channels.

The first requirement can be achieved with any number of low-pass filters. The second requirement, while perhaps not as obvious, can be achieved by designing a Nyquist filter and oversampling the channelizer output [28]. This yields $\hat{x}\left(n\right)\approx cx\left(n-k\right)$ from (5) where the error can be controlled by the filter design.

**Figure 1:**
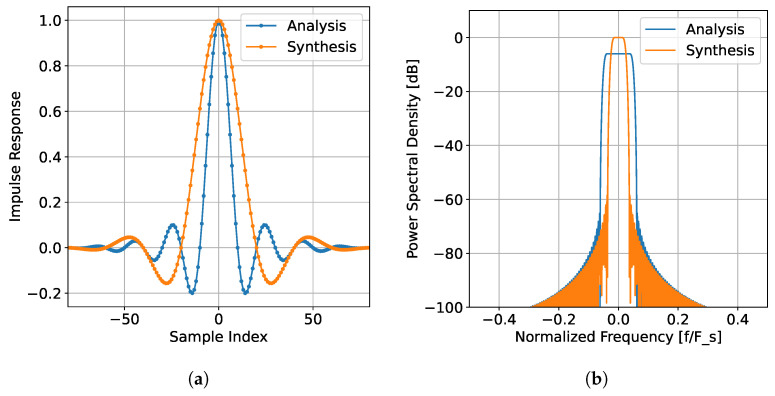
Polyphase filterbank (PFB) channelizer responses with M=20 channels, a prototype filter semi-length of 4, and a stop-band suppression of 60 dB: (**a**) impulse response of $h_{0}\left(n\right)$ and $g_{0}\left(n\right)$ and (**b**) spectral response of $h_{0}\left(n\right)$ and $g_{0}\left(n\right)$.

An example of a channelizer prototype filter’s impulse and spectral responses is shown in Figure 1, below, for M=20 channels. It is noticed that because the analysis channelizer is oversampled by a factor of 2 on its output, its prototype filter has twice the bandwidth of that of the synthesis filter. Specifically, the pass-band cut-off frequency for $h_{0}$ is $F_{s}/M$ while the pass-band cut-off for $g_{0}$ is $F_{s}/2M$.

Once the signal is channelized, the outputs $R_{i}\left(k,m\right)$ undergo digital processing—either singular value decomposition (SVD) reconstruction or MVDR—to remove the interference. Afterwards, the next step is to synthesize the *N* newly processed signals $Y_{i}\left(k,m\right)$ back into a single signal $v_{i}\left(n\right)$.

### 2.5. SVD Reconstruction

One way to remove the nuisance signals is to take advantage of their relatively high power spectral density in comparison to SOI. This is achieved by taking the SVD of a block of samples across all sensors for the *m*-th sub-band, removing the high-powered singular values, and reconstructing the sample block.

The SVD of any matrix **A** can be represented as

(15) $$A=U\Sigma V^{H}$$

where ${\left(\cdot \right)}^{H}$ denotes the Hermitian transpose operation. If **A** is an n×m matrix with rank *r*, then **U** is an n×r matrix, and **V** is an m×r matrix. The diagonal matrix **Σ** is an r×r matrix, whose diagonals are the singular values (σ). The singular values are strictly positive and are in descending order of energy (e.g., $\sigma_{1}>\sigma_{2}>\ldots >\sigma_{r-1}>\sigma_{r}$). Another way of viewing the singular values is as the positive square roots of the eigenvalues of $A^{H}A$.

The SVD of **A** can also be expressed as

(16) $$A=\sum_{i=1}^{r}\sigma_{i}u_{i}v_{i}^{H}$$

where $u_{i}$ is the *i*-th column vector of **U**, and $v_{i}$ is the *i*-th column vector of **V** [30].

Another way to consider **A** is as a linear combination of basis functions. According to [31], the first *r* columns of **U** are an orthonormal basis for the column space of **A**. Likewise, the first *r* columns of **V** are an orthonormal basis for the row space of **A** while the last m−r columns of **V** are an orthonormal basis for the null space of **A**. In the case of digital phased arrays, the null space is, in actuality, represented as the noise space of the observation across antenna elements.

After decomposition, the high-powered nuisance signals are associated with the low-ordered (high-powered) singular values, and noise is related to the highest order (lowest power) singular values. For the proposed SVIRA, two thresholds are used to remove noise and interference. As only the singular values that fall between the two thresholds are kept, the upper threshold is chosen to remove the interfering signals while the lower threshold is chosen to remove the noise. The recomposed matrix $\tilde{A}$ is then

(17) $$\tilde{A}=\sum_{i\in R}\sigma_{i}u_{i}v_{i}^{H}$$

where R is the set of singular value indices to be kept. If selected appropriately, $\tilde{A}$ should contain SOI, minimal noise, and no interference. Therefore, choosing appropriate threshold values is important in order to maximize SOI and limit interference. The threshold selection process is discussed in greater detail in Section 2.7.

### 2.6. Signal of Interest Enhancement

To increase the chance of decoding SOI, SOI is enhanced by combining the sensors in such a way as to maximize the coherent gain in matrix **A**. This can be achieved using the following optimization:

(18) $$c=\underset{\tilde{c}}{arg max}\left({\tilde{c}}^{H}A^{H}A\tilde{c}\right)$$

where c represents the columns of **A**, which contains the data for each sensor. To reach a nontrivial solution (i.e., prevent ∥c∥→∞), *c* is constrained such that ∥c∥=1. With this constraint in place, (18) can be solved using the Rayleigh quotient as follows:

(19) $$\lambda_{\min}\left(A^{H}A\right)\leq \frac{c^{H}A^{H}Ac}{c^{H}c}\leq \lambda_{\max}\left(A^{H}A\right)$$

where $\lambda_{k}$ represents the *k*-th eigenvalue of the square matrix $A^{H}A$. The lower bound of (19) is achieved by the eigenvector corresponding to the minimum eigenvalue of $A^{H}A$. Likewise, the upper bound is achieved by the eigenvector corresponding to the maximum eigenvalue of $A^{H}A$ [32].

In this way, the coherent gain of the columns of **A** is maximized by using the coefficients that maximize the Rayleigh quotient as weights to combine the columns of **A**, which relates to the sensors. To ensure that the gain is continuous from frame to frame, the phase of each sensor is adjusted before combination. That is to say, the elements of the weighting vector c are allowed to be complex, but the solution to (18) is therefore unique only to a phase ambiguity (e.g., if $\hat{c}$ is a solution to (18), then so is $\hat{c}e^{j\theta }$ for all angles θ). Computation of the eigenvalues for (19) using computer systems often results in a phase ambiguity that can be problematic when combining finite-length observations. As such, the ambiguity is resolved by rotating all elements of c by the same phase such that arg{c(0)}=0.

### 2.7. Singular Value Selection

While absolute thresholds work when the range of SVs is known, it is inconvenient and inefficient to calculate them all before setting the thresholds. This calculation must be performed not only for every new packet and interference combination, but it is also necessary for every change in channelizer parameters (i.e., number of channels and block size). Therefore, a relative threshold selection process is preferred. Since the PSD of SOI is assumed to be flat, the noise floor can be estimated as the lowest—weakest-powered—singular value (SV) and can be used as a reference point for thresholding. Since the thresholds are relative, they are more robust to changes in channelizer parameters, and the range is mainly dependent on the SNR of the packet.

However, when there are more signals present a in sub-band than there are sensors in the array, the weakest SV no longer contains only noise. In this case, the lowest SV represents the signal as well as the noise subspace, which can cause the SV to fluctuate wildly. In order to compensate for fluctuations in the lowest singular value, SVIRA observes SV magnitudes across frequencies for a given block of samples and takes the tenth percentile of them as a reference point. The tenth percentile is assumed to be aggressive enough to be indicative of noise but not any signals that could be present in the SV. This results in flat thresholds across frequency, which in turn allow for proper suppression of the nuisance signals.

Another problem case is when SOI and an interferer share a similar DOA. In this case, SOI and the interference share eigenspace, and due to the nature of the SVD, the interference will appear in the same singular value as SOI. Therefore, a trade-off between introducing a notch in SOI for that particular sub-band or allowing a certain amount of interference to remain must be considered. In simulation, it is determined that it is better to notch SOI and accept a small amount of ISI; otherwise, SOI cannot be decoded. Furthermore, the amount of ISI introduced by notching one sub-band in that particular case is not enough to prevent successful decoding from happening.

### 2.8. Configuration Order

**Figure 2:**
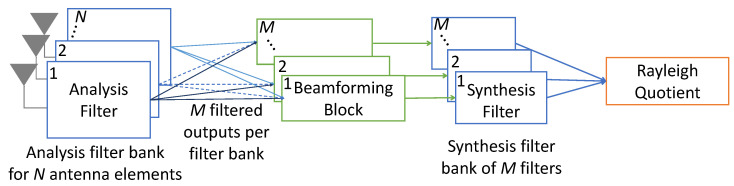
Block diagram of overview of singular value interference removal algorithm (SVIRA).

The three main components—channelizer, SVD reconstruction, and combination—can be arranged in different configurations to make up SVIRA. There are two main configurations that SVIRA could take: Combination Before Synthesis and Combination After Synthesis. Combination After Synthesis (Figure 2) is characterized by combining the sensor data and using the Rayleigh quotient, after synthesizing across frequencies with the synthesis filterbank and interference removal. The general procedure for this configuration with an *N*-sensor array is as follows:

1. The data are passed through *N* identical PFB analysis filterbanks with *M* channels.
2. Each of the *M* filtered outputs across the *N* sensors are then processed using SVD reconstruction to remove the interference.
3. The resulting data are synthesized using a bank of *M* synthesis filters.
4. Finally, the data are combined across sensors using the Rayleigh quotient.

**Figure 3:**
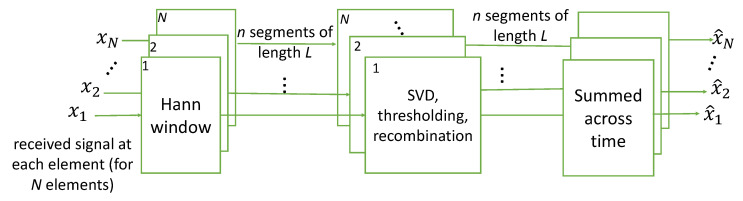
Inside the singular value decomposition (SVD) reconstruction beamforming block.

A block diagram of the general procedure is outlined in Figure 2, where the beamforming block contains the SVD reconstruction block from Figure 3.

A more detailed explanation of the process is as follows:

1. The complex valued matrix **R** has the dimensions N×k, where *N* is the number of antenna elements (or sensors), and *k* is the number of samples in the buffer.
2. The matrix **R** is filtered through an analysis filterbank defined by an *M*-band channelizer, whose output is oversampled by a factor of 2. This process outputs a sequence of matrices $C_{m}$ for m=0,1,2,…,M−1, where *m* corresponds to the band number. Each matrix $C_{m}$ has the dimensions N×2L, where 2L is the block size after decimation.
3. Each matrix $C_{m}$ is windowed by a periodic, *L*-point Hann window into framed segments, which have a segment overlap of 50% and sample length *L*. That is to say, for every sensor $c_{i}$ with the indices 1:k, the newly framed counterpart is $c_{f,i}=c_{i}\left[fL:\left(f+1\right)L-1\right]$, where *f* is the frame index, which continues to iterate until it segments the entire matrix $C_{m}$. The total number of frames is heavily dependent upon *L* and the amount of overlap.
4. For each frame, the resulting matrix $A_{m}$—for band number m=0,1,2,…,M−1—has the size L×N. As noted by its size, $A_{m}$ contains the framed data from all *N* sensors.
5. The matrix $A_{m}$ is then processed—and interfering signals are removed—using SVD reconstruction.
6. The frames are linearly combined to reconstruct each ${\hat{C}}_{m}$, which has the same shape as $C_{m}$ (N×2L). Given the type of window and the amount of overlap, $C_{m}$ would be perfectly reconstructed in the time domain if singular values had not been removed from $A_{m}$. As such, the newly reconstructed ${\hat{C}}_{m}$ is $C_{m}$ with the nuisance signals mostly, if not completely, removed.
7. Each ${\hat{C}}_{m}$ is synthesized through its own synthesis filter (*M* synthesis filters in total) to produce the matrix $\hat{R}$, which has the shape N×k.
8. The data from all of the sensors in $\hat{R}$ are combined, using weights defined by the Rayleigh quotient, into a single vector $\tilde{r}$, which is *k* samples long.
9. The probability of packet error is analyzed by attempting to decode the packet in $\tilde{r}$.

Another configuration is Combination Before Synthesis, which combines across sensors before using a synthesis filter to recombine across frequencies. However, there is a coherence loss associated with this configuration. While the channelizer is designed for near-perfect reconstruction, it does not take combining across antenna elements into account. Therefore, if a bank of synthesis filters is used to first reconstruct the received data on each element, and the data are combined across sensors after synthesis, then the coherence of the signals is better maintained, thus providing a better result. As such, in this paper, SVIRA only refers to the Combination After Synthesis configuration.

### 2.9. Simulated Single Packet Scenarios

This section investigates the performance of SVIRA on a single packet in three scenarios: (1) three tonal interference sources, (2) one narrowband interference source, and (3) one wideband interference source. In these examples, the SOI is set to impinge upon the 4-element uniform circular array (UCA) from 0° in the azimuth. SOI has an SNR of 3 dB, and all interference sources have an SINR of 10 dB. Even without the interference, the SNR of the packet is not high enough to decode. Thus, in addition to removing the interference, SOI requires additional gain enhancement, which SVIRA achieves by coherently combining the antenna elements.

#### 2.9.1. Scenario 1: Three Interference Sources

**Figure 4:**
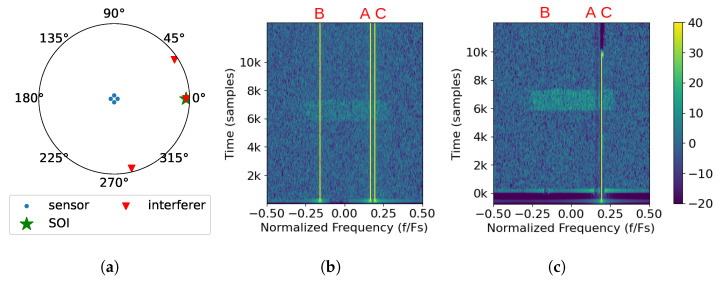
Example of the SVIRA process operating on a single frame with three interferers (Scenario 1) with an signal-to-interference-plus-noise ratio (SINR) of 10 dB; an signal-to-noise ratio (SNR) of the packet of 3 dB, M=100, 2L=64; and SVD thresholds set as −1 dB to 15 dB: (**a**) the array configuration; (**b**) spectral input to SVIRA as seen by a single element; (**c**) spectral output of SVIRA.

A scenario is considered when there are 3 interference sources: A, B and C. Sources A, B, and C impinge upon the array at an azimuth of 33°, 284°, and 0°, respectively (see Figure 4a). Furthermore, each source produces one interfering tone with the respective center frequencies of 0.166 $F_{s}$, −0.157 $F_{s}$, and 0.195 $F_{s}$ as seen in the spectrum received at the first element in Figure 4b. After undergoing the SVIRA, the output spectrum in Figure 4c shows that interfering signals A and B are successfully removed while interfering signal C is reduced in power but not removed. As such, SOI cannot be decoded. The observations below can be made from Figure 4.

1. There is a processing delay in the output incurred by both the PFB channelizer as well as the windowing function, which is visible in the output plot.
2. The majority of SOI experiences 6 dB enhancement by coherently combining the antenna elements as made evident by its higher power level in the output plot; however, because of interference C, it cannot be decoded.
3. Interferences A and B can be successfully removed with minimal impact on SOI, but interference C cannot. While all of the interfering signals are frequency-separable and thus can be processed relatively independently, since interference C comes from the same DOA as SOI, the interference cannot be separated from SOI. This is due to them both sharing the same eigenspace and thus singular values. The only way to potentially decode SOI is to allow the sub-band containing interference C to be notched out, which will introduce some ISI and performance degradation.
4. Due to how close the DOA of interference A is to the DOA of SOI, the power of SOI is lowered in the particular sub-band where the interference is removed.
5. Unlike with interference A, SOI does not show signs of reduced power in the same band where interference B is removed. This is due to the DOA of the interference being sufficiently removed from that of SOI, so they do not share singular values (or similar eigenspace). Thus, the interference can be removed without visibly impairing the power of SOI.

#### 2.9.2. Scenario 2: Narrowband Noise Interference Sources

**Figure 5:**
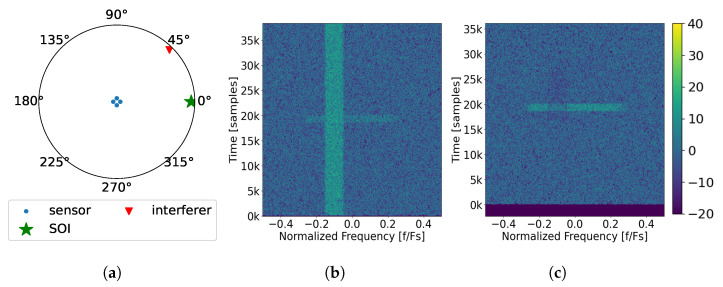
Example of the SVIRA process operating on a single frame with a narrowband interferer (Scenario 2) with an SINR of 4 dB; an SNR of the packet of 3 dB, M=300, 2L=64; and SVD thresholds set as −1 dB to 7 dB: (**a**) the array configuration; (**b**) spectral input to SVIRA as seen by a single element; (**c**) spectral output of SVIRA.

In addition to removing tones, SVIRA is able to remove narrowband noise interference. A scenario is considered where there is a narrowband noise signal that occupies 10% of the band and arrives at the previously established array from 45° in the azimuth as shown in Figure 5a. Similar to previous scenarios, SOI has an SNR of 3 dB and a DOA of 0°, but the interference has an SINR of 4 dB. The narrowband noise has a normalized center frequency of −0.100 $F_{s}$, which can be seen in the spectrum from the first element, Figure 5b, along with SOI. After using SVIRA, the narrowband noise is successfully removed, and SOI can be decoded. The following observations can be made from Figure 5:

1. Overall, the majority of the SOI has a 6 dB improvement.
2. The narrowband noise interference is successfully removed; however, like with interference A from Scenario 1, in the sub-bands where the narrowband noise interference used to be, SOI is slightly reduced in power. This is likely due to the DOA of the interference being close enough to SOI’s DOA such that SOI and the interference share singular values and have similar eigenspaces.

#### 2.9.3. Scenario 3: Wideband Noise Interference Sources

**Figure 6:**
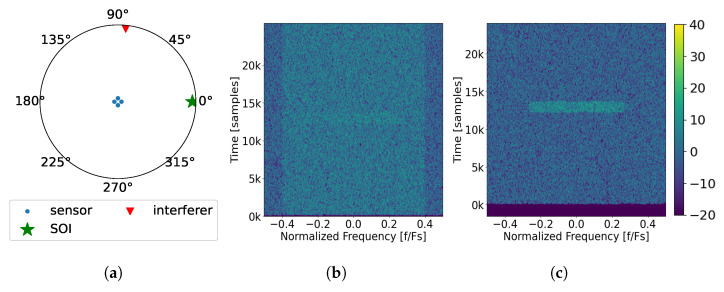
Example of the SVIRA process operating on a single frame with a wideband interferer (Scenario 3) with an SINR of 2 dB; an SNR of the packet of 3 dB, M=200, 2L=64; and SVD thresholds set as −1 dB to 8 dB: (**a**) the array configuration; (**b**) spectral input to SVIRA as seen by a single element; (**c**) spectral output of SVIRA.

Finally, a scenario is considered when there is wideband noise interference. As shown in Figure 6a, the array configuration is the same as before, and the wideband interference arrives at the 84° azimuth, with an SINR of 2 dB. When observing the spectrum as seen by the first element in Figure 6b, the interference is shown to be centered in frequency and occupies 80% of the band, completely covering SOI. After applying SVIRA to the samples, the spectrum output displayed in Figure 6c reveals SOI, which can be decoded without any of the wideband interference. The following observations can be made from Figure 6:

1. Once again, SOI is shown to have the same 6 dB enhancement as in previous scenarios.
2. The wideband interference is successfully removed with minimal disturbance to SOI as it arrives from an entirely different direction than SOI.

While these scenarios prove that SVIRA can handle multiple interference types (tones, narrowband noise, and wideband noise), for the sake of simplicity, only tonal interference is considered in experimentation.

### 2.10. MVDR

As a point of comparison to SVIRA, we consider MVDR when the array configuration and DOA of SOI are known. In this case, MVDR beamforming can also be used to enhance SOI and remove interference while operating on channelized sub-bands as described above. This beamformer creates weights that minimize the amount of interference and noise while maintaining a distortionless response in the DOA of SOI, thus maximizing the SINR of the input. However, since MVDR requires knowledge of SOI’s DOA and the array configuration, it is not always feasible to implement.

The algorithm to implement MVDR, as described by [33], is as follows. At any instant *k*, the output of the beamformer y(k) can be represented as

(20) $$y\left(k\right)=w^{H}x\left(k\right)$$

where x(k) is the input at instance *k*, ${\left(\cdot \right)}^{H}$ indicates the Hermitian transpose, and **w** is a vector of weights determined by the algorithm. As stated previously, the purpose of MVDR is to maximize the SINR of the input signal, which is

(21) $$\mathrm{SIN}R≜\frac{E[|w^{H}s{|}^{2}]}{E[|w^{H}\left(i+n\right){|}^{2}]}=\frac{\sigma_{s}^{2}{\left|w^{H}a\left(\theta_{s}\right)\right|}^{2}}{w^{H}R_{i+n}w}$$

where **a**$\left(\theta_{s}\right)$ is the steering vector, $\sigma_{s}^{2}$ is the power in SOI, **i** and **n** are the interference and noise vectors, respectively, and E[·] signifies the expectation. $R_{i+n}$ is a matrix of the expectation of the covariance of the interference plus noise.

(22) $$R_{i+n}≜E\left[\left(i\left(k\right)+n\left(k\right)\right){\left(i\left(k\right)+n\left(k\right)\right)}^{H}\right]$$

Now that the SINR is defined, the maximization problem is such that the interference and noise are minimized and the input signal is fixed. This can be expressed as the following optimization problem

(23) $$\min_{w}w^{H}R_{i+n}w\;\mathrm{such}\;\mathrm{that}\;w^{H}a\left(\theta_{s}\right)=1$$

whose solution is

(24) $$w_{\mathrm{MVDR}}=\alpha R_{i+n}^{-1}a\left(\theta_{s}\right)$$

where $\alpha =1/a^{H}\left(\theta_{s}\right)R_{i+n}^{-1}a\left(\theta_{s}\right)$ and ${\left(\cdot \right)}^{-1}$ indicates the matrix inversion.

**Figure 7:**
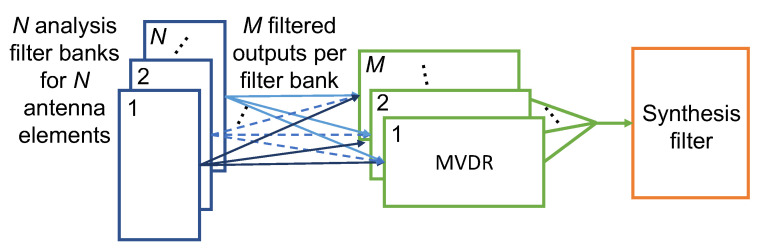
Block diagram of the minimum variance distortionless response (MVDR) process with a PFB channelizer.

As a point of comparison to SVIRA, MVDR is used in conjunction with the channelizer. The block diagram for this process can be seen in Figure 7. MVDR is used in place of SVD reconstruction, and since MVDR inherently combines across antenna elements, Rayleigh combination is not needed. Therefore, the modified process, starting at step 5 is as follows:

5. MVDR is performed on the matrix **A**_*m*_. The result is the vector ${\hat{a}}_{m}$ of length 2L.
6. The frames are then linearly combined to reconstruct $\tilde{r}$.
7. The probability of packet error is analyzed by attempting to decode the packet in $\tilde{r}$.

### 2.11. Simulated Comparison to MVDR

In order to assess the efficacy of the proposed algorithm, independent data packets are generated at a particular SNR coincidental with a 2-dimensional, uniform circular array (UCA) with a spacing of 0.3 wavelengths. It is important to note that the array does not have to be in this specific geometry for the SVD reconstruction process to work. Theoretically, the proposed SVD reconstruction method could work with any array configuration as it does not use the configuration information. Interference sources are represented as tones, each generated at a random frequency and transmitted from a random angle in the azimuth. For the purpose of simplicity, interference from various points in elevation is not considered; all signals are transmitted from an elevation of 0°. The resulting signals from each antenna element are processed with two algorithms. The first is our proposed SVIRA, which is described above. The second is MVDR processing with a PFB channelizer. It should be noted that in both cases, the channelization steps are used; otherwise, the number of interfering sources would exceed the array’s degrees of freedom. We use packet error rate (PER) as a metric for comparison over SINR as it is easily measured in a wireless environment and provides a more complete picture of the overall distortion.

**Figure 8:**
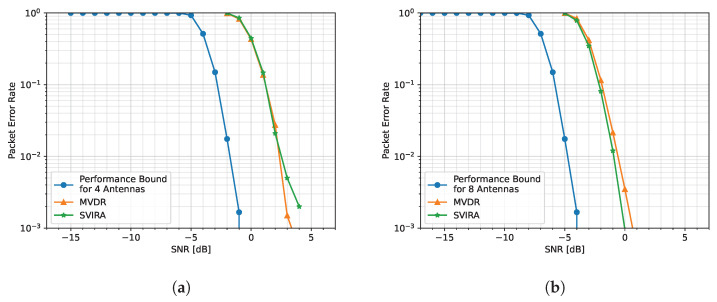
Comparison of MVDR and SVIRA with 8 interference sources for a (**a**) 4-element array and an (**b**) 8-element array; M=200, 2L=64, SVD thresholds are −1 dB to 15 dB, and the MVDR threshold is 5 dB.

The performance bounds for both Figure 8a,b are determined by simulating transmitting and receiving packets in an additive white Gaussian noise (AWGN) channel with no interference and no beamforming. The PER curves for the 4-element array case are shown in Figure 8a. It is noticed that while MVDR and SVIRA are not able to achieve the performance bound (due to the distortion of SOI when forming nulls at nearby angles), the two curves have a similar PER performance in comparison to each other. Similarly, the 8-element array PER curves are shown in Figure 8b. Once again, the curves for MVDR and SVIRA have roughly the same performance. Even without the knowledge of the array configuration, SVIRA has a comparable simulation performance to MVDR, which heavily relies on a priori knowledge. While MVDR has been shown to have significant performance loss if the array configuration is not known, SVIRA does not experience the same loss as it does not rely on knowledge of the array configuration.

### 2.12. Key Technical Contributions

This work addresses the limitations of both traditional (required precise knowledge of the array configuration and the DOA of SOI known a priori) and blind beamforming (accurate estimation of the covariance matrix, the total number of signals in the band known a priori, SOI possessing certain statistical properties, etc.) with a novel algorithm for multi-element receivers, in which narrowband nuisance signals are isolated in frequency, and the restriction of precise knowledge of the element positions is relaxed. In fact, the only restrictions on the sensor elements are that they are frequency-locked (albeit with a potentially unknown phase offset), and they are relatively close to one another in the sense that wavefront propagation across the array can be approximated with a simple carrier phase shift. The proposed algorithm is designed with the following properties in mind:

1. Works with legacy waveforms.
2. Operates without precise antenna configuration knowledge.
3. Can remove more interference sources than degrees of freedom afforded by the phased array with frequency separability.
4. Does not require estimation of interference number or types.
5. Able to achieve a similar PER performance as MVDR.

A brief comparison of beamforming algorithms can be found in Table 1 including an overview of computational complexity for MVDR, MUSIC, other eigen-based methods, and SVIRA. As is the case for all algorithms, computational complexity is dominated by computing and operating on the N×N cross-covariance matrix, such as computing its inverse or eigenvalue decomposition. The complexity for SVIRA is similar but requires the additional step of decomposing the wideband stream into *M* narrowband sub-bands. This can be computed efficiently using a polyphase filterbank channelizer. While the resulting singular value decomposition operates on each of the *M* channels, they are running at a rate of approximately 1/M of the input. As such, the complexity of SVIRA is commensurate with existing beamforming algorithms subject to the choice of the channelizer size *M*.

## 3. Experimentation

### 3.1. Hardware Setup and Description

**Figure 9:**
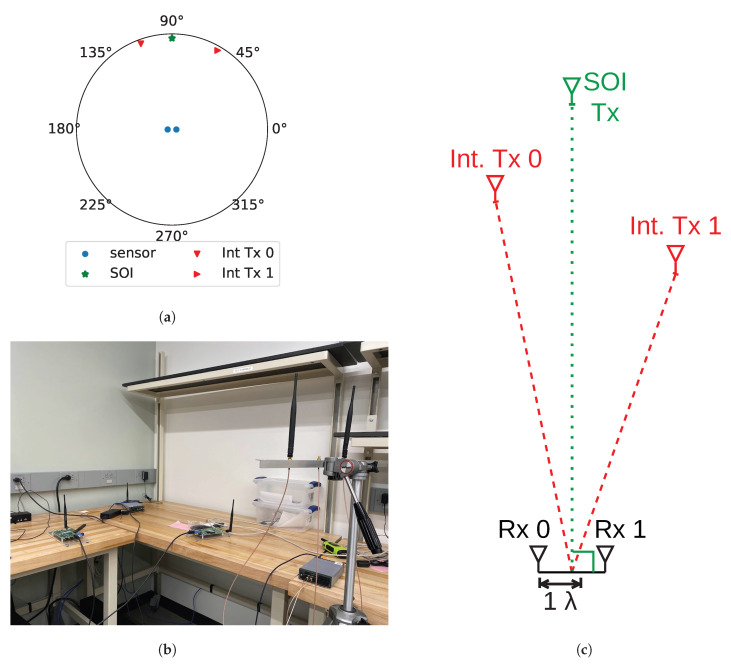
The lab setup: (**a**) depiction of the relative positions of the 2-element receiver, interference transmitters, and the transmitter for the signal of interest (SOI); (**b**) picture of the setup in the lab; (**c**) diagram of relative distances between transmitters and receivers in wavelengths.

Experimentation was conducted in a laboratory with dense multi-path effects and sources of interference. Additionally, since the lab is a shared environment, during certain data capture periods, other researchers are present in the room and may have been moving around. A picture of the actual lab setup is shown in Figure 9b, and a diagram of the antenna locations is shown in Figure 9c. As seen in Figure 9b, the main transmitter is composed of a universal software radio peripheral (USRP) E-320 [34] and a dipole antenna. SOI is transmitted at a center frequency of 2.205 GHz and a sample rate of 100 kHz. However, since there is considerable local oscillator (LO) leakage, the antenna is off-tuned to compensate.

The receiver array is placed approximately 154 cm away from the SOI transmitter as displayed in Figure 9c. The array consists of two antennas and each antenna is connected to one of the receiver ports on a USRP E-320. The centers of each antenna element are approximately 1.6 wavelengths apart. Additionally, each element of the array is approximately equidistant from the transmitter of SOI to achieve a value as close as possible to equal receiver gain at each antenna element.

It is important to note that the array does not have to have this specific geometry for SVIRA to work. Theoretically, the proposed method could work with any array configuration so long as the elements are frequency-locked and relatively close together as it does not consider the positions of the individual elements. Additional elements could be added to create a larger array, which would increase the antenna gain, but this also increases the computational complexity (see Table 1). With that being said, this method is limited in the same way as other phased-array techniques. If the elements are too far apart, the wavefront propagation can no longer be approximated with simple carrier phase shifts, and as such, additional factors such as time delays must be considered when processing.

The interference signals are generated using antennas connected to two USRP B-210s. The first interference transmitter (Int. Tx. 0) is located 131 cm away, and the other (Int. Tx. 1) is 100 cm away. During testing, either one or both of the B-210s are used to transmit uniformly spaced tones of a given number. Although only tonal interference is verified using hardware, other types of interference could also be verified using the same hardware in the same configuration. Since other interference types have been shown to be removed in simulation, it is likely that they can also be removed from captured data from a lab experiment.

### 3.2. Data Collection Setup

For each dataset, there are two distinct consecutive transmissions: one capture contains just SOI while the second contains SOI and interference. Transmission A only concerns the SOI packets, whose transmission gain sweeps from a high to low gain in 1 dB steps. During this transmission, the receiver is turned on first and then the SOI transmitter is activated to send packets. Neither of the interference emitters is on during this transmission sequence, so the resulting data should just be SOI on top of the background spectral environment. This should allow for an accurate depiction of the current spectral environment at the time of the transmission. Once Transmission A is completed, Transmission B immediately follows. Transmission B transmits SOI packets at the same gain values as Transmission A; however, this time, intentional interference in the form of tones is added. The transmission sequence is a follows: The interference emitter or emitters are turned on and transmit continuously throughout the data capture. Next, the receiver is switched on, and finally, the SOI transmitter is allowed to transmit packets.

Since the spectral environment could change in a given moment due to the other equipment in the lab, it is best to transmit the second transmission sequence in quick succession. Additionally, as the lab is shared and captured data are collected at different times on different days, any of the antennas could have moved between transmissions, resulting in different DOAs and distances between the transmitters and the receiver array. Therefore, it is important to always transmit both transmissions A and B consecutively.

Transmissions A and B allow for a direct comparison of the proposed process with and without relatively high SNR interference in a real-world environment. Simple PER curves are once again used as the preferred method to analyze the efficacy of the proposed algorithm on the captured data. By embedding the transmission gain value into the header of the packet, it is simple to generate PER curves based on the known transmission gain. However, when comparing the captured data to the simulation, using transmission gain as a basis is no longer a viable option since it cannot be directly compared to the known SNR values of the simulated packets. Therefore, a process is needed to transform the known transmitted gain values into approximate SNR values, so the captured and simulated data can be compared side by side.

### 3.3. SNR and Gain Calibration

In order to directly compare the captured hardware results’ theoretical bounds, a method of estimating SNR is devised. The transmission gain of each packet is used as a basis to estimate the SNR because it is known. Since the transmission gain and SNR of the packet should have a linear relationship, only one gain–SNR relationship pair needs to be found in order to calculate the SNR for the rest of the transmission gains. Once a gain–SNR reference pair is determined, the *i*-th SNR associated with the *i*-th gain can be calculated using

(25) $${\mathrm{SNR}}_{i}\left[\mathrm{dB}\right]={\mathrm{gain}}_{i}+\left({\mathrm{SNR}}_{\mathrm{ref}}-{\mathrm{gain}}_{\mathrm{ref}}\right)$$

As such, it is important that the estimated reference SNR is as accurate as possible.

**Figure 10:**
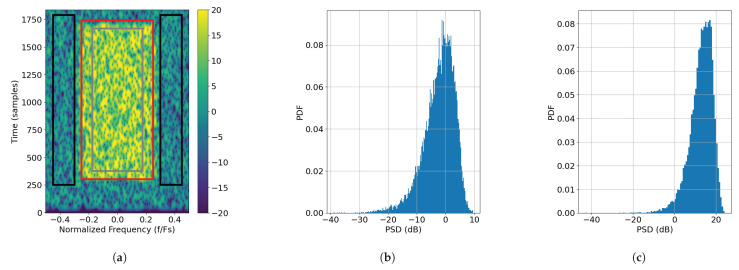
SNR calibration example: SNR of the packet is 15 dB, and the noise floor is 0 dB. (**a**) The hlpower spectral density (PSD) of the snippet is divided by 4 bounding boxes: the black bounding boxes contain only noise; the red bounding box is where the packet is assumed to be, and a smaller bounding box is drawn in gray to gain a more accurate signal-plus-noise PSD. The probability density of the (**b**) noise and (**c**) signal-plus-noise powers are calculated to validate the ordered statistic choice for SNR estimation.

To estimate the SNR of a single packet, first, the index of the beginning of the packet is found, which is used to create a snippet of the data file that isolates the packet. Then, the PSD of the snippet is taken and a bounding box is drawn around the packet, which is represented in red in Figure 10. Using the principle of exclusion, a smaller bounding box containing the signal-plus-noise PSD is drawn in gray, and the two bounding boxes in black are created to solely contain the PSD of the noise. The signal-plus-noise power and noise power can both be determined using ordered statistics on the PSD values in the appropriate bounding boxes. Once the signal-plus-noise power and noise power are estimated, the SNR can be approximated using

(26) $$\mathrm{SNR}\left[\mathrm{linear}\right]=\frac{\mathrm{signal}\;\mathrm{power}-\mathrm{noise}\;\mathrm{power}}{\mathrm{noise}\;\mathrm{power}}$$

Thus, it is important to achieve a reasonably accurate estimation (<0.1 dB) of both the signal-plus-noise power and the noise power.

The use of ordered statistics attempts to mitigate the influence of unknown signals in the background of the snippet. In this example, the median noise power is −1.5 dB, and the median signal-plus-noise power is 13.7 dB, which gives an SNR estimate of 15.0 dB. On the other hand, the mean of the noise power is 0.0 dB, and the mean of the signal-plus-noise power is 15.2 dB, which gives an SNR estimate of 15.0 dB. Despite the estimated SNR values being the same, the approximated signal-plus-noise powers and noise powers are different by 1.5 dB. By estimating at different SNR and noise floor values, a 1.5 dB bias is found when using the median as the ordered statistic. Therefore, the mean is used as the ordered statistic of estimation. This choice is validated by looking at the probability density of the PSD values of the noise (Figure 10b) and signal-plus-noise (Figure 10c), which are both skewed to the right.

**Figure 11:**
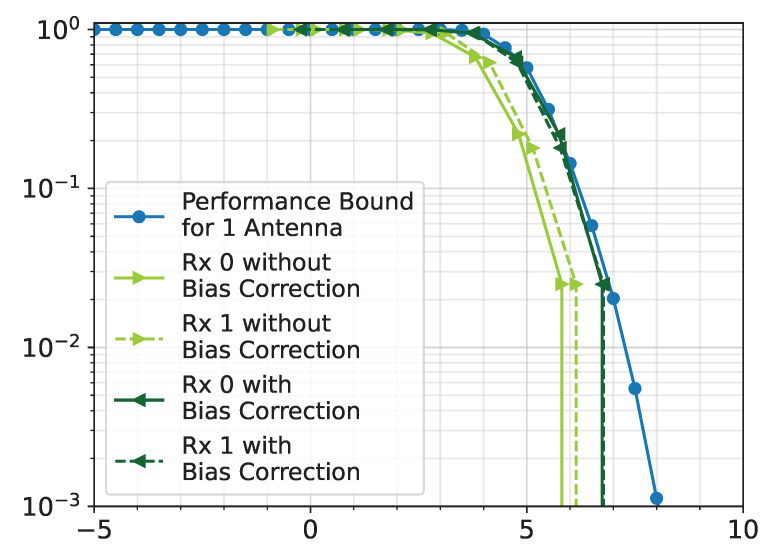
Packet error rate (PER) curves for Rx. 0 and Rx. 1 from File 27 (with and without bias correction).

**Figure 12:**
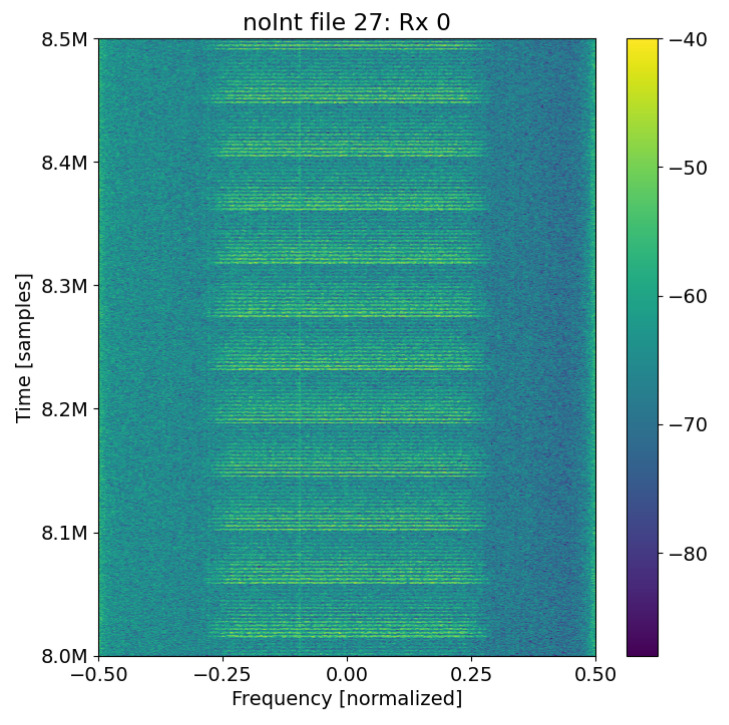
Snippet from captured data File 27, which shows the uneven noise floor.

Although this method is able to accurately estimate the SNR of simulated packets, it does have some bias when estimating the SNR of captured packets. It can be observed how Rx. 1 and Rx. 0 without the bias correction fall about 1 dB away from the simulated performance bound in Figure 11. Since the difference between the two is consistent, an inherent bias of 1 dB is surmised. This could in part be due to the background noise not being flat across the band as seen in Figure 12. It is noticed that the noise floor on the left edge of the band is higher than that on the right edge. The average power on the left edge is −64.5 dB while it is −67.9 dB on the right edge, which means there is around a 3.4 dB discrepancy in the noise floor from the left to the right side of the band. It is possible that the front end of the receiver has a small frequency-dependent response, and the signal itself could have a small frequency-dependent slope. Despite averaging the two edges, the resulting SNR still has a small bias as seen in Figure 11.

The bias is corrected using the error vector magnitude (EVM) of the estimated packets. Since the EVM is a measurement of the overall distortion of the symbols, it not only takes noise distortion into account, but it also considers IQ mismatch, nonlinearity, thermal noise, channel distortion, carrier leakage, etc. Thus, EVM should not be used to accurately estimate SNR, but it can be useful to correct the bias. The bias removal procedure is as follows:

1. Find the EVM of each packet.
2. Estimate the SNR of each packet.
3. For each packet, determine the difference between the EVM and estimated SNR.
4. Take the median of the differences as the bias.
5. Add the bias to the estimated SNR.

Once the bias is removed, the Rx. 0 and Rx. 1 align with the performance bound (as displayed in Figure 11) as expected.

## 4. Experimental Results

The first capture (File 8) contains no interference, so its results are used as a baseline performance for the other two captures. The second capture (File 27) includes eight interfering tones from Emitter 0. Finally, the last capture (File 41) has 13 interfering tones: 6 from Emitter 0 and 7 from Emitter 1. Only Transmission A is considered for File 8, which contains 800 sweeps. Likewise, File 41 contains 800 sweeps for Transmission A, but it also has 1000 sweeps for Transmission B. Each sweep for both Files 8 and 41 contains one packet per transmitted gain level, which ranges from −8 dB to −22 dB, inclusive, in 1 dB steps (total of 15 steps). Thus, Files 8 and 41 each contain 12,000 packets worth of data for Transmission A, and File 41 has 15,000 packets worth of data for Transmission B. File 27 contains 200 sweeps for Transmission A and 500 sweeps for Transmission B. Each sweep also has 15 steps total, but its transmission gains range from −10 dB to −24 dB, inclusive, in 1 dB steps. As such, there are 3000 packets in Transmission A and 7500 packets in Transmission B.

In order to ensure the simulated data used for comparison are analogous to the captured data, three simulated datasets are created to emulate the captured transmissions. However, the channel for each simulated transmission is AWGN so as to establish a baseline ideal performance. Simulated File 4 is created to simulate Transmission A. File 4 receives 1000 sweeps of 14 packets, whose SNR varies from −5 dB to 8 dB, inclusive, by 1 dB. Simulated File 5 is generated to emulate the circumstance of File 27’s Transmission B. There are eight interfering tones set to come from one emitter (roughly the same DOA as Emitter 0), and 1000 sweeps containing packets whose SNR ranges from −5 dB to 9 dB, inclusive, are received through an AWGN channel. Finally, simulated File 6 is constructed to emulate the data received in File 41. There are 1000 sweeps of 14 packets, whose SNR starts at 8 dB and sweeps down to −5 dB in 1 dB steps. Additionally, there are 13 interfering tones generated across the band: 6 coming from a similar DOA as Emitter 0 and 7 coming from a similar DOA as Emitter 2.

A summary of the parameters used to process each captured file is shown in Table 2. Likewise, Table 3 shows the parameters used to process the generated files. It should be noted that each of the simulated files is processed using the same parameters as their captured counterparts. The parameters used for each file are deemed the optimal parameters for that particular file based on performance sweeps conducted on the captured files.

### 4.1. No Interference Source

**Figure 13:**
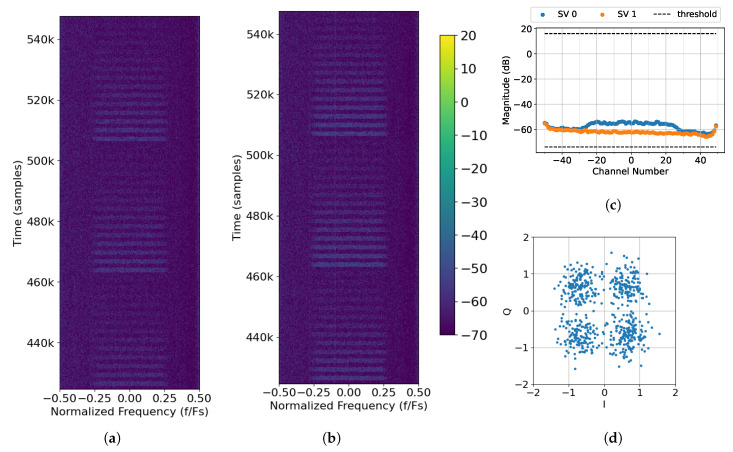
Processing captured data File 8 (No Interference Source Case) with *M* = 96, 2L = 64, and thresholds of −1 dB to 80 dB: (**a**) raw spectrum as seen by Rx. 0; (**b**) output of SVIRA; (**c**) SVs across frequencies for samples at 458k–461k; (**d**) constellation of Packet 49, which has a gain of −12 dB.

Figure 13 depicts the data from File 8 before, during, and after they are run through the proposed algorithm. A snapshot of the spectrum as captured by Rx. 0 can be seen in Figure 13a. Since File 8 is only concerned about Transmission A, approximately three full sweeps can be seen in the spectrogram without any interference. Therefore, the raw data from sensors Rx. 0 and Rx. 1 can be combined without the need to threshold for interference removal. This is achieved by setting the thresholds wide (−1 dB to 80 dB) and inputting the raw data into the algorithm. By setting the thresholds wide, most of the SVs should be kept as they all fall within the thresholds (see Figure 13c), so there should be a 3 dB performance gain—associated with combining two antenna elements—at the output of the algorithm. A snapshot of the spectrum of the output is shown in Figure 13b, where there is indeed an increase in the power of each packet. While it is difficult to determine if there is indeed a 3 dB improvement by looking at the spectrograms, it becomes clear when looking at the PER.

**Figure 14:**
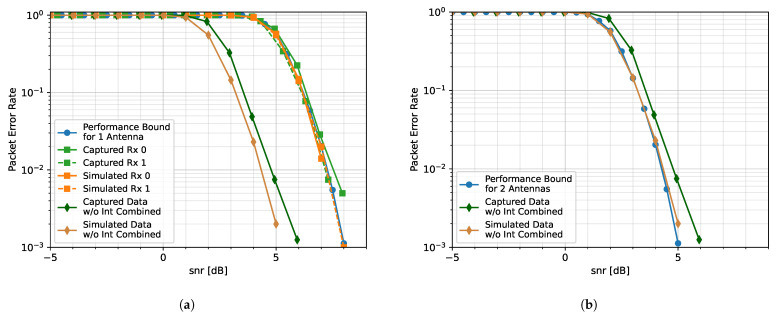
Comparison of the PER curves for packet decoding under an additive white Gaussian noise (AWGN) channel with no interference to the simulated performance bound: (**a**) a 1-element receiver case; (**b**) a 2-element receiver case.

Figure 14a compares the performance of the proposed algorithm on simulated data and the captured data from File 8. It should be noted that the decoded raw data from Rx. 0 and Rx. 1 for both captured and simulated data align with the expected performance bound for a single antenna. This indicates that the SNR of captured data is properly estimated and there are no major issues with the hardware. When the two simulated raw sensors are combined, there is a 3 dB improvement from the raw data, which can be seen when comparing the PER of the combined sensors (orange diamonds) to the raw sensors (orange squares). Similarly, there is a roughly 3 dB improvement when the raw, captured sensors Rx. 0 and Rx. 1 are combined. This improvement is made more evident when the simulated and captured combined sensor curves are directly compared to the performance bound of an array with two antennas in Figure 14b. It should be noted that the combined simulated sensors follow the performance bound exactly, as expected. However, there is a small performance difference between the combined captured Rx. 0 and Rx. 1 and the performance bound, which can be attributed to imperfections introduced by the hardware such as channel distortion, LO leakage, etc. Additionally, there are a few signals in the spectral background (seen in Figure 13b, when zooming in) that are also enhanced during the process and prevent successful decoding at lower SNRs.

### 4.2. One Interference Source Case

**Figure 15:**
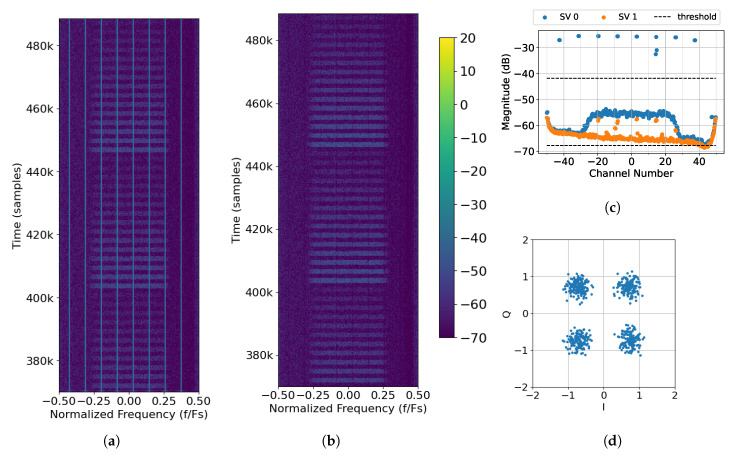
Processing captured data File 27 (One Interference Source Case) with *M* = 370, 2L = 128, and thresholds of −1 dB to 15 dB: (**a**) raw spectrum as seen by Rx. 0; (**b**) output of SVIRA; (**c**) SVs across frequencies for samples at 464k to 488k; (**d**) constellation of Packet 62, which has a transmission gain of −12 dB.

Next, a case is considered when interference is introduced. Interference Tx. 0 transmits eight, evenly spaced tones across the band as seen in Figure 15a. All eight tones can be clearly seen in the singular values (Figure 15c) and are removed by virtue of the upper threshold. The resulting spectrum (Figure 15b) no longer has visible interference. Once the interference is removed, the packets can be decoded. The constellation for successfully decoded Packet 62 is shown in Figure 15d. It is noticed that while there is some distortion to the symbols overall, all of the symbols are tightly grouped in the appropriate quadrant.

**Figure 16:**
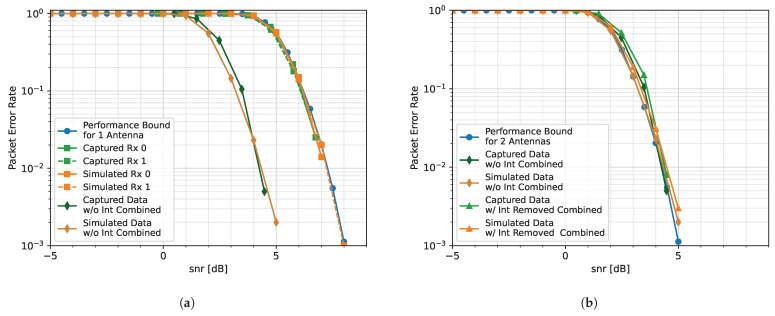
PER curves for the One Interference Source Case, emitting 8 tones: (**a**) PER curves for Transmission A; (**b**) PER curves for Transmission B.

Once again, when no interference is transmitted, both simulated and captured Rx. 0 and Rx. 1 align with the performance bound for one antenna (Figure 16a), which indicates that the SNR calibration for this capture is correct. When the data from the respective arrays are combined, there is a 3 dB improvement. It should be noted that the curve representing the combined captured elements (green diamonds) is very similar to that of the simulated elements (orange diamonds). The performance difference between the two is 0.5 dB at most. It is possible that the difference in performance between this case and the one presented in Figure 14a is due to the spectral background on the given day or the position of the antennas.

After introducing interference and removing it with the proposed algorithm, the resulting PER curves are closely aligned with the performance bound for two antennas (Figure 16b). While there is some performance loss between the simulated combined without interference curve (orange diamonds) and the combined with interference removed curve (orange triangles), it is very small. Therefore, the algorithm is capable of producing results that are nearly as good as receiving packets on a two-element antenna array without any interference. There exists a similar performance loss between the captured combined without interference curve (green diamonds) and the combined with interference removed curve (green triangles), which indicates that the algorithm is capable of achieving a similar level of success with data captured from over the air.

### 4.3. Two Interference Sources Case

**Figure 17:**
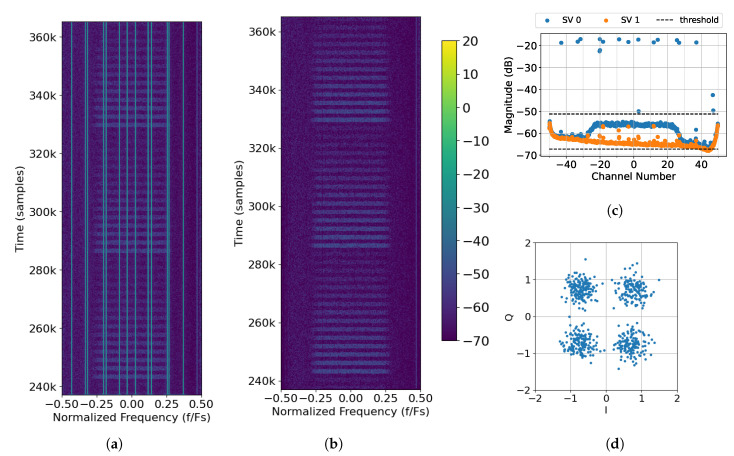
Captured data from File 41 (Two Interference Sources Case) with *M* = 800, 2L = 320, and thresholds of −1 dB to 15 dB: (**a**) raw spectrum as seen by Rx. 0; (**b**) output of SVIRA; (**c**) SVs across frequencies for samples 237k–265k; (**d**) constellation of Packet 64, which has a gain of −12 dB.

Finally, a case is considered when there are two interference sources. In this case, Tx. 0 transmits six evenly spaced tones, and Tx. 1 transmits seven. The spectrum showing the location of the tones and the received packets is shown in Figure 17a. The singular values and thresholds are displayed in Figure 17c. It is noticed that the upper threshold is chosen to be as tight as possible to the pedestal that represents SOI in order to remove as much interference as possible. While the interference is removed, there is a null at around −0.18 $F_{s}$ (see Figure 17b). This null, and others like it, causes ISI, which introduces distortion that is visible in the constellation. We observed that the constellation clusters in Figure 17d are not as tight as they were in Figure 15d.

**Figure 18:**
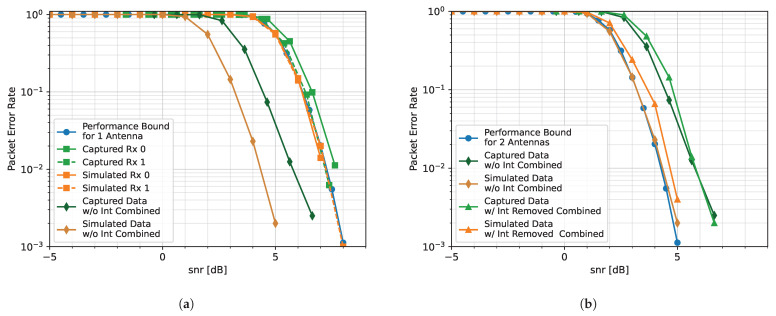
PER curves for the Two Interference Sources Case, emitting a total of 13 tones: (**a**) PER curves for Transmission A; (**b**) PER curves for Transmission B.

In this capture, the captured Rx. 0 has a worse PER performance than the captured Rx. 1 (Figure 18a). The captured Rx. 0 has an approximate 0.5 dB performance loss in comparison to the performance bound for one antenna and Rx. 1. This is likely due to the position of the array at the time of data capture, which resulted in the received antennas having different effective gains. Although the PER curves for each captured element are similar to the simulated elements, their combined curves have a performance difference of almost 2 dB.

This performance difference is also seen when the interference is removed (Figure 18b). In the simulated case, the PER curve (orange triangles) is within 0.5 dB of that of the combined without interference curve (orange diamonds), which is aligned with the performance bound for two antennas. This suggests the algorithm, with the current parameters, has difficulty removing all of the interference without impacting the integrity of the packet. Similarly, in the hardware case, when the interference is removed, the resulting PER (green triangles) has, at most, a 0.5 dB performance degradation in comparison to the combined case with no interference (green diamonds). Since both the simulated and captured cases involving interference removal perform nearly as well as when the elements are combined when no interference is present, it is likely that the 2 dB performance difference between the simulated and captured cases is due to the difference between the simulated and captured scenarios.

There are several possibilities that could account for the degradation in PER performance. One issue is the uneven noise floor (see Figure 17c), which can be mitigated by introducing equalization before synthesizing. Another possibility could be that there are more sources of interference within a sub-band than there are degrees of freedom. To mitigate this issue, additional elements could be added to the array or the number of sub-bands can be increased. In a similar vein, if the interference lies in the transition band between channels, it gets aliased and potentially “sneaks through” the thresholds. This can be mitigated by using a different number of sub-bands. One final issue could be that SOI is in a similar eigenspace as the interference. Unfortunately, since SIVRA is similar to an eigen-based beamformer, it is not able to distinguish between the interference and SOI. By suppressing the interference, SOI is also suppressed.

## 5. Discussion

In this paper, a novel beamforming approach to interference removal in spectrally congested environments is proposed which does not rely on knowledge of the antenna locations, orientations, or phase offsets. Furthermore, SVIRA operates independently of the waveform of interest (no pilots or special correlation properties are used), outside the assumption of a flat power spectral density. Captured data from the two-element array laboratory experiment verify that the proposed beamforming approach achieves a packet error rate within half a decibel of the theoretical performance bound of receiving packets in AWGN. Although SVIRA performs about 1 dB worse than the performance bound when there are two sources of interference, SVIRA is able to achieve PER performance similar to the case when no interference is present by removing the narrowband interferers, with a performance loss of at most 0.5 dB. Thus, SVIRA can remove interference in such a way that there is minimal PER performance loss in comparison to when there is no interference present in the band.

## Figures and Tables

**Table 1:** Comparison of beamforming algorithms.

	Requires Knowledge of Array Configuration	Requires Estimation of SOI DOA	Requires Estimation of Number of Signals	Algorithm Complexity per Input
MVDR	Yes	Yes	No	$O(N^{2})$
MUSIC	Yes	Yes	No	$O(N^{2})$
Eigen-based	No	No	Yes	$O(N^{2})$
SVIRA	No	No	No	$O(NM\log M+MN^{2})$

**Table 2:** Parameters used for the data files captured using hardware.

	Number of Sweeps	Transmission Gain Range per Sweep (dB)	Number of Tones	Number of Interference Sources	M	B	Thresholds (dB)
File 8 (Trans. A)	800	−8 to −22	0	0	96	64	−1, 80
File 27 (Trans. A)	200	−10 to −24	0	0	96	64	−1, 80
File 27 (Trans. B)	500	−10 to −24	8	1	370	128	−1, 25
File 41 (Trans. A)	800	−8 to −22	0	0	96	64	−1, 80
File 41 (Trans. B)	1000	−8 to −22	13	2	800	320	−1, 15

**Table 3:** Parameters used for the generated (simulated) data files.

	Number of Sweeps	SNR Range per Sweep (dB)	Number of Tones	Number of Interference Sources	M	B	Thresholds (dB)
File 4	1000	−5 to 8	0	0	96	64	−1, 80
File 5	1000	−5 to 9	8	1	370	128	−1, 25
File 6	1000	−5 to 8	13	2	800	320	−1, 15

## Data Availability

The raw data supporting the conclusions of this article will be made available by the authors upon request.

## Abbreviations

The following abbreviations are used in this manuscript:

*M*	the number of sub-channels in channelizer
*N*	the number of antenna elements (“sensors”)
*h*_0_	the channelizer prototype filter
2*L*	the block size for processing SVD, consistent with *L* for Hann window
*x*_*i*_(*n,m*)	*i* is the element index; *n* is the time index; *m* is the channel index
AWGN	additive white Gaussian noise
CCI	co-channel interference
CMA	constant modulus algorithm
CNN	convolutional neural network
CSV	composite steering vector
dB	decibels
DFT	discrete Fourier transform
DOA	direction of arrival
EM	electromagnetic
EVM	error vector magnitude
FCC	Federal Communications Commission
FFT	fast Fourier transform
IoT	Internet of Things
IQR	interquartile range
ISI	intersymbol interference
ISM	industrial, scientific, and medical
LO	local oscillator
LMS	least mean-squares
MUSIC	multiple signal classification
MVDR	minimum variance distortionless response
OFDM	orthogonal frequency-division multiplexing
PDF	probability density function
PER	packet error rate
PFB	polyphase filterbank
PSD	power spectral density
RQ	Rayleigh quotient
QPSK	quaternary phase-shift keying
RFFE	radio frequency front end
RFID	radio frequency identification
RLS	recursive least-squares
SCORE	spectral self-coherent restoral
SDR	software-defined radio
SIC	successive interference cancellation
SINR	signal-to-interference-plus-noise ratio
SNR	signal-to-noise ratio
SOI	signal of interest
STAP	space–time adaptive processing
SV	singular value
SVD	singular value decomposition
SVIRA	singular value interference removal algorithm
UCA	uniform circular array
USRP	universal software radio peripheral
